# Research on coupling optimization of carbon emissions and carbon leakage in international construction projects

**DOI:** 10.1038/s41598-024-59531-4

**Published:** 2024-05-10

**Authors:** Zhiwu Zhou, Ying Wang, Julián Alcalá, Víctor Yepes

**Affiliations:** 1 Hunan Provincial Key Laboratory of Intelligent Protection and Utilization Technology in Masonry Artifacts, School of Civil and Environmental Engineering, Hunan University of Science and Engineering, Yongzhou, 425006 Hunan China; 2 Chongqing Vocational College of Public Transportation, Chongqing, China; 3 Institute of Concrete Science and Technology (ICITECH), Universitat Politècnica de València, Valencia, Spain

**Keywords:** Construction industry, Environmental impact, Carbon trading, Model evaluation, Environmental sciences, Civil engineering

## Abstract

Due to the rapid economic development of globalization and the intensification of economic and trade exchanges, cross-international and regional carbon emissions have become increasingly severe. Governments worldwide establish laws and regulations to protect their countries' environmental impact. Therefore, selecting robustness evaluation models and metrics is an urgent research topic. This article proves the reliability and scientific of the assessment data through literature coupling evaluation, multidisciplinary coupling mathematical model and international engineering case analysis. The innovation of this project's research lies in the comprehensive analysis of the complex coupling effects of various discrete data and uncertainty indicators on the research model across international projects and how to model and evaluate interactive effects accurately. This article provides scientific measurement standards and data support for governments worldwide to formulate carbon tariffs and carbon emission policies. Case analysis data shows that the carbon emission ratio of exporting and importing countries is 0.577:100; the carbon trading quota ratio is 32.50:100.

## Introduction

Due to the increasingly severe carbon emission reduction challenge caused by global climate change and the pressure brought by climate deterioration, countries in the world are formulating relevant policies and developing new emission technologies to reduce the climate impact while proposing preventive measures such as border carbon leakage and border carbon adjustments (BCAS), etc. Carbon leakage refers to the phenomenon caused by the transfer of carbon-intensive products from countries with strict carbon constraints to countries with loose carbon constraints due to the asymmetry of carbon costs between different regions. Carbon leakage causes: consumers in developed countries to use imported products with low price but high carbon content to replace domestic products with high prices and low carbon content^1^. Carbon adjustment refers to relevant policy measures taken by regions and countries to cope with the impact of carbon leakage, including taxing the carbon content of imported products at the border^2^.

Based on the 10-year carbon emission study of the Regional Comprehensive Economic Partnership (RCEP), Torres-Machí drew the following conclusions^3^: the liberalization of trade has dramatically reduced the carbon emission intensity of developed countries and eased the carbon emission burden of developing countries. With the implementation of the carbon tariff, carbon emissions have been transferred from developing countries to developed countries. In contrast, the transfer of welfare costs caused by climate policy is just the opposite. However, the total emissions are still on a growth trend. The increase in international trade has made countries gain economic benefits from multilateral trade, brought significant environmental impact to countries of origin and destination countries. For instance, in Sino-US trade, with the growth of China's trade volume, China has become a net exporter of embodied carbon emissions^4–6^.

The global carbon dioxide (CO_2_) emissions in 2018 were 25.6 peta-grams, of which China, the United States, and Western Europe accounted for 29.6%, 16.4%, and 11.8%, respectively^7^. Research shows that the reduction of carbon emission intensity and the optimization of industrial structure can inhibit the increase of carbon emissions, which can be understood as the emission reduction competition between technological innovation and the changes in production structure and consumption patterns^8^.

Theoretically, carbon leakage involves four channels: investment, competitiveness, energy prices, and offshore emissions. The amount of carbon contained in global trade shows a rapid growth trend. In 2004, the estimated value was 4–6 GtCO2 (accounting for approximately 20–30% of global emissions), and in 2006 it reached 7–8 GtCO2 (accounting for approximately 25–35%)^9^. Sato^10^ Research surveys indicate that specific carbon flows at the national level remain highly uncertain for most countries and years. When comparing the reported results of existing annual survey studies, it will be found that there are apparent inconsistencies in the data, and existing problems undermine the robustness of the quantification. Michael Grubb^11^ reviews the state of knowledge on international CO_2_ emissions transfers, namely that solutions must be inherently evolutionary, testing options and “feeling the rocks” in a complex minefield of conflicting opinions and international interests, and that one-size-fits-all policies cannot be implemented. Therefore, it is significant to accurately and scientifically assess carbon leakage in international trade and investment projects based on industry characteristics. The potential carbon leakage scale and risk of developing countries are higher than those of developed countries and gradually determine the trend of global climate governance. Research shows that the carbon leakage rate of developing countries is 5–20% and remains around 14%^12^. Some countries try to achieve environmental benefits by implementing of carbon tariff policies. However, research shows that carbon tariffs will have more harm than sound effects on the economy, welfare, and international trade of collectors and holders and impact carbon-intensive enterprises. This leads to changes in the country's overall economic output and welfare level, especially the economy of export-oriented countries^13^. It is stipulated in *American Clean Energy and Security Act* (ACESA), revised in 2009, that it will impose a carbon tariff on the imported carbon-intensive products from countries that do not implement carbon emission reduction policies from 2020 or the imported products with lower carbon emission standards than the United States^14^. Shui and Harriss studied the environmental impact of Sino-US trade from 2002 to 2007^15^, concluding that the embodied energy range is 7–11 exajoule, accounting for 12–17% of China's total energy consumption. CO_2_ range is 400–800 Mt, accounting for 8–12% of China's total emissions.

At present, in accordance with the primary trade principles stipulated by the World Trade Organization: the basic interpretation of the National Treatment Principle (NT, Article 1) and the Most Favored Nation Principle (MFN, Article 3): NT prohibits products from countries other than its own; MFN prohibits its own country from discriminating against other countries’ goods of other countries rather than those of third countries. It is necessary to avoid the conflict between climate and trade systems damaging global trade and climate agreements^16^. The Americas and Europe have taken some measures on carbon emissions. They will gradually increase the mandatory tariff measures on carbon emissions in international trade and implement carbon emission reduction policies focusing on low carbon industries^17^. However, carbon tax policies implemented by most countries violate the basic principles of the WTO, leading to vicious competition in the trade war.

It is agreed in the *Paris Agreement* that the world should achieve carbon neutrality by 2050; the global emissions should be reduced by more than 50% in 2030; temperature rise is limited to < 2 ℃, and the optimal target is 1.5 ℃. However, infrastructure construction affects more than 50% of global emissions. Research predicts that global infrastructure investment will reach USD 94 trillion by 2040, and carbon emissions will grow rapidly and continuously^18^. Among them, the investment in international investment projects increased rapidly. In 2016, the global revenue of the top 250 international contractors reached USD 468.12 billion; in 2020, the commercial value of the global construction industry reached USD 12.7 trillion, accounting for 15% of the global GDP^19^.

Contractors mainly obtain international construction projects through the following ways: engineering procurement construction (EPC model); competitive tender. The former means that the investor selects the bid-winning contractor with the lowest price through comprehensive assessment and multiple rounds of negotiation and that the contractor shall complete the design, procurement, construction, and operation tasks per the contract. The latter means that the best bid winner will be determined based on the tender offer and expert assessment and that the contractor shall complete the project construction task according to the contract^20^. EPC project is characterized by a complex process, numerous subcontractors, a long construction period and fixed contract price, and its main risks are quality, cost, supplier, owner, and schedule because of high uncertainty in the international market and complex EPC process^21^. The research case herein is the EPC model.

How to accurately assess the carbon leakage and emission in international investment projects? The literature survey shows that this field needs to be improved and strengthened (see “Literature review” section for detailed analysis); in particular, there needs to be a case study and analysis of search results of the construction industry. The research objectives of this article are as follows: (1) The article establishes a complete mathematical assessment model and research framework for international engineering carbon emissions and carbon leakage across countries and regions through research and innovation. (2) Apply the internationally recognized traceable industry chains databases such as Ecoinvent and PSILCA global supply chain and the sustainable standard assessment system (OpenLCA15.0) specified by ISO14040 (International Organization for Standardization) and 14044 to analyze the carbon leakage emission footprint and data accurately. (3) Select China-Indonesia transnational significant bridge cases built and operated as the analysis object and use accurate and practical data analysis to verify the robustness and scientificity of the theoretical evaluation model.

This paper attempts to contribute to existing research in the following areas:

1. Complete literature survey and analysis of the current research status and main research directions in this field through Citespace visual bibliometric coupling analysis and Wolfram Mathematica13.2 powerful mathematical calculation modelling, supplementing and enriching the research vacancies in this field.
2. This article uses two ISO standard framework systems (ISO14040 and 14044) and assessment systems (ISO sets seventeen sustainable development goals) to conduct research and accurately select multiple traceable life-cycle databases (Ecoinvent, PSILCA and Agri-footprint, etc.) Analyze the loss of fossil materials in carbon leakage and its environmental impact on carbon emissions.
3. Analyze the impact of carbon leakage data on international projects through research models and physical cases and discuss urgent scientific issues in the sustainable environmental optimization design of cross-international projects.
4. The published literature lacks detailed research on carbon leakage cases in the construction industry; the research methods of published articles mainly focus on applying economic theory and interview questionnaire methods to discuss the carbon leakage mechanism, supporting policies and theoretical analysis of border carbon taxes. More solid, innovative cross-international theoretical models must be used to accurately study the causes of quantified carbon leakage with accurate data and a series of life-cycle impacts.
5. Quantitatively analyze the coupling factors of carbon leakage from a macro perspective through actual case studies and improve the theoretical model-data-optimization systematic, innovative research on carbon leakage in this field. Provide researchers in the same field and direction with a better understanding and application of models for analysis. The trajectory of carbon leakage in other industries sets the paradigm.
6. This research model and accurate data analysis can provide measurement standards and scales for countries worldwide to accurately assess carbon emissions and carbon leakage taxes and fees across the international construction industry and avoid unnecessary economic sanctions and friction due to the lack of scientific assessment methods.

The article is divided into six sections. The first section (Introduction) introduces the current situation of global carbon emissions and leakage; The second section (Literature review) analyses the gaps of international engineering in carbon leakage research; The third section (Methodology) establishes the theoretical model framework of the study; Section four (Results): Process analysis of international project cases; Section five (Discussion) discusses the causes and emissions of carbon leakage and emissions in international projects; The sixth section (Conclusion) the important research contents, existing defects and future research directions of the article.

## Literature review

The United States, the European Union and China account for 61% of global GDP and 43% of commodity imports and exports. Therefore, the three parties' Carbon Border Adjustment Mechanism has great potential in reducing emission leakage, sector competitiveness or regional distribution, especially in energy-intensive consuming industries^22^. In order to solve the above problems, the EU proposed a carbon border adjustment mechanism as "a climate measure that should prevent the risk of carbon leakage and support the EU's increased ambition in climate mitigation while ensuring compatibility with the WTO". Due to the quantification of asymmetric carbon, there needs to be more substantive scientific guidance on how emissions are sufficient to justify carbon policy actions. The EU emissions trading system has experienced multiple fluctuations. The industries most affected by carbon leakage are carbon-intensive trading commodities in the construction industry, such as steel, cement, and aluminium^23^. Zhou's^24^ recent research focuses on the impact of carbon tariffs on international trade, which, in reality, affects global supply chain companies and low-carbon-regulated countries. More accurate data on the impact of carbon tariffs on global supply chains and carbon emissions is needed. The significant studies conducted have not provided directional data support and decisions on the carbon tariff response strategies required by exporting countries to reduce social welfare damage. In order to explain these problems, it is necessary to construct a set of global supply chain decision-making research models adapted to different regulatory systems around the world so that governments can analyze the essential impact of carbon emissions on carbon tariffs, social welfare and consumer surplus. Eskander^25^ analyzed the impact of climate legislation in 111 countries on trade-related carbon leakage from 1996 to 2018 and concluded that the positive and negative are roughly similar. The core reason is that the impact on trade-related emissions is not statistically significant. In short, there is a lack of evidence of trade-related carbon leakage. Combining the above high-quality (Q1) research to accurately assess international trade carbon leakage is an urgent scientific issue.

In order to further expand the scope of research, this article uses CiteSpace 6.1.R2 software to conduct visual econometrics coupling analysis of published literature, grasp subject hot spots and development trends in the same field, predict future subject development directions, and present scientific knowledge structures and laws in a visual way and distribution. The research principle is mathematical and statistical quantitative analysis^26^.

The research literature data come from the Web of Science (WOS) database^27^. Advanced retrieval is adopted, and the retrieval keywords are Construction industry (CI), Carbon leakage (CL), International trade (IT), and international engineering (IE). The retrieval conclusion is as follows: CI + CL + IT = 7 pieces; CI + CL = 41 pieces; CI + IT = 1473 pieces; CL + IT = 419 pieces; CL + IE = 949 pieces; CI + CL + IE = 8 pieces and CL + IT + IE = 67 pieces. Due to the insufficient number of other retrieved literature, 419 and 949 groups of data were finally selected for bibliometric coupling analysis.

**Figure 1:**
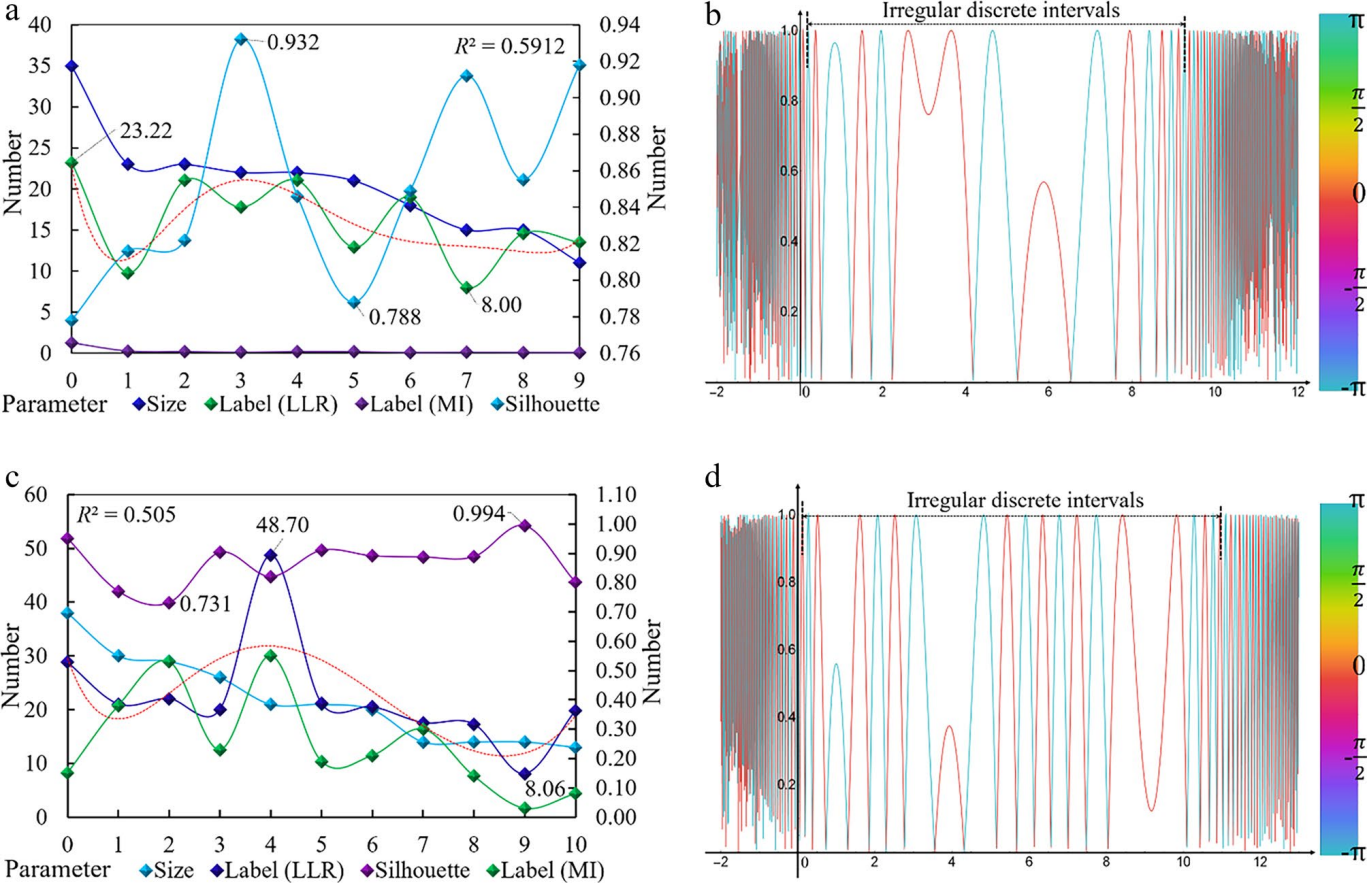
Clustering coupled analysis of database. (**a**) Analysis of keyword clustering network map (CL + IT). (**b**) Discreteness analysis of burst term (CL + IT). (**c**) Analysis of keyword clustering network map (CL + IE). (**d**) Discreteness analysis of burst term (CL + IE).

For 419 articles (time interval: 1993–2022, Modularity = 0.5941 > 0.5; Silhouette = 0.8411 > 0.7; Har monic mean (Q, S) = 0.6963 > 0.5. The three assessment indexes prove that the clustering structure is significant; the clustering data are very compelling; the structure is accurate); the keyword clustering network atlas analysis is carried out, and ten groups of core burst terms (2010–2018) are obtained. Two groups of linking words are North–south trade and Carbon trading. Thirteen groups of the fifty keywords are related to trade, from regional and international trade to theoretical system research and regulations and policies. Twelve groups are related to carbon leakage, focusing on carbon emissions, carbon markets, and carbon tariffs (Fig. 1a). According to the analysis data obtained, the research on international trade and carbon leakage mainly published in the time interval focuses on manufacturing and agriculture. There are many studies on the European carbon markets. Figure 1b analyzes the change of the LLR curve value of the core burst terms from 2010 to 2018, lineally showing that the published articles are irregularly discrete in the range of 0 to 10 and continuously discrete in the range of −∞,0 and 10, + ∞. $\mathit{sin}\theta$ the model shows that there are research literatures in this field before 2010 and after 2018, and the number of published literatures shows a downward trend.

For 949 articles (time interval: 1991–2022, Modularity = 0.6699 > 0.5; Silhouette = 0.8635 > 0.7; Harmonic mean (Q, S) = 0.7544 > 0.5. The three assessment indexes prove that the clustering conclusion is superior), 55 groups of linking words (2012–2017) are obtained. Among them, 12 groups are directly related, accounting for 21.8% of the total. The research focuses on carbon leakage, carbon storage, and carbon capture, involving materials, electric energy, and theoretical research (Fig. 1c). Eleven core burst terms are related to carbon leakage and CO_2_ leakage stress. Through data analysis, the CL + IE-type clustering conclusion can better reflect the research status and direction in this field. Figure 1d shows the discreteness, showing that the published articles are regularly discrete in the range of − ∞, − 0.2 and 11, + ∞, and the number of the research literature is increasing to decreasing. The published articles are irregularly discrete in the range of − 0.2–11, with the time interval spanning 6 years. Comparing the two research methods, the former has a comprehensive research scope, a significant time, and a lack of core content. The latter is more concentrated and closer to the research content of this paper but has a narrow time range, few results, and a prominent core research field.

### Comprehensive evaluation

Through the bibliographic coupling analysis, it is concluded that the research on carbon leakage has started on a global scale, with a focus period of 2010 to 2018, involving the fields of food, materials, agriculture, and a small amount of fossil energy. Agriculture and materials account for a large proportion, and the construction industry only accounts for 11.63%. The research focuses on carbon emissions, carbon storage, and carbon trading. Some of the achievements are relevant to the construction industry. However, the in-depth research on carbon leakage of international investment projects is blank and needs to be supplemented and strengthened.

## Methodology

It is widely used internationally to calculate the embodied carbon of trade: single regional input–output (SRIO), bilateral trade input–output (BTIO), and multi-regional input–output (MRIO). The method review concludes that MRIO is the most appropriate method for embodied emissions in national-level trade. The problem is the need for more transparency in the pioneering methodology and use of databases and the assessment of many uncertain factors^28^.

In this section, the theoretical framework and the mathematical model for assessing carbon leakage of international investment projects are established. Due to the particularity of international investment projects and the coupling effect of multiple factors, there are many uncertainties in establishing the theoretical system, such as natural environment, social environment, project participants, project contracting mode, procurement and transportation of materials and equipment, labour force, local laws and regulations, land acquisition, etc.^29^.

Based on the characteristics of the industry model and the existing uncertainties, the following assumptions are made: (1) Chinese contractors account for 60% of the Asian contracting market, and their time performance is better than that of other continents. In order to guarantee the timeliness, the critical equipment and primary raw materials of the study case are purchased in the exporting country to ensure fast and convenient delivery and avoid delays in material transportation and customs clearance^26^. (2) The theoretical framework does not consider the impact of project-related uncertainty and delay factors, including human resources, production interface, procurement, design, environment, natural, social, cultural, technical, and legal factors^30^. (3) The assessment of embodied carbon emissions of the labour force is carried out following the provisions of ISO and the local laws and regulations of the project, with low carbon, environmental protection, and energy conservation as the measurement criteria^31^. (4) The theoretical system is established to study and assess the carbon leakage in the whole life cycle of preliminary design, raw material extraction and manufacturing, transportation, construction, operation and use^32^. (5) The integrative transportation system is adopted to ensure dynamic optimization under supply conditions. The criteria of rationality, efficiency, energy consumption reduction and environmental pollution reduction are followed^33^. 6. The carbon quota of exporting and importing countries is not considered in the carbon leakage assessment, and the carbon leakage data are analyzed based on the actual emissions and economic and trade quota in the supply chain to ensure the transparency and accuracy of the methodology^34^.

**Figure 2:**
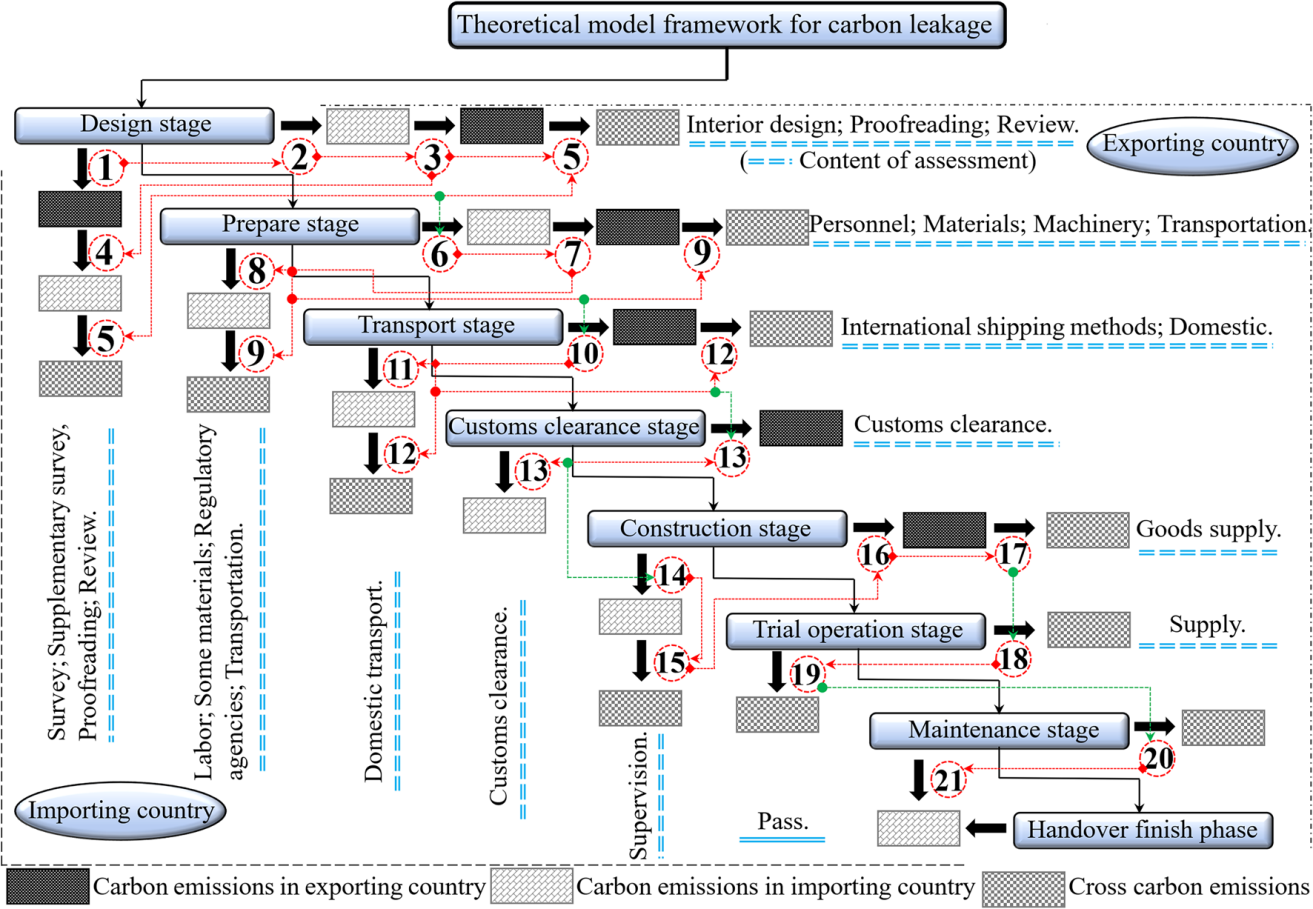
Theoretical model framework system for carbon leakage.

Figure 2 shows the international investment project assessment system and carbon leakage framework model. The number represents the node; the red line represents the route and direction; the green line represents the optimal node and direction across the system stage; the two blue dotted lines represent the key work content of each stage. From the analysis of the model system, it can be found that carbon leakage has uncertainties in the carbon trading system. Most engineering-related carbon-intensive industries are concentrated in exporting countries, while there is a higher risk of carbon leakage for import-oriented countries. Therefore, it is necessary to establish a systematic assessment model to analyze the carbon leakage data of international investment projects.

The uncertainties of the supply chain system include the strict degree of regulation of carbon emissions by exporting and importing countries; differences in emission price; the uncertainty of emission cost; imbalance of trade; carbon emission intensity of the economy; energy, and trade specialization. In order to avoid the interference of the above factors, the research and analysis data are all traceable life cycle inventory data and software analysis data, and the actual carbon emission data are referred. In addition, the current carbon trading market price for the carbon economy and trade are referred^32^.

**Figure 3:**
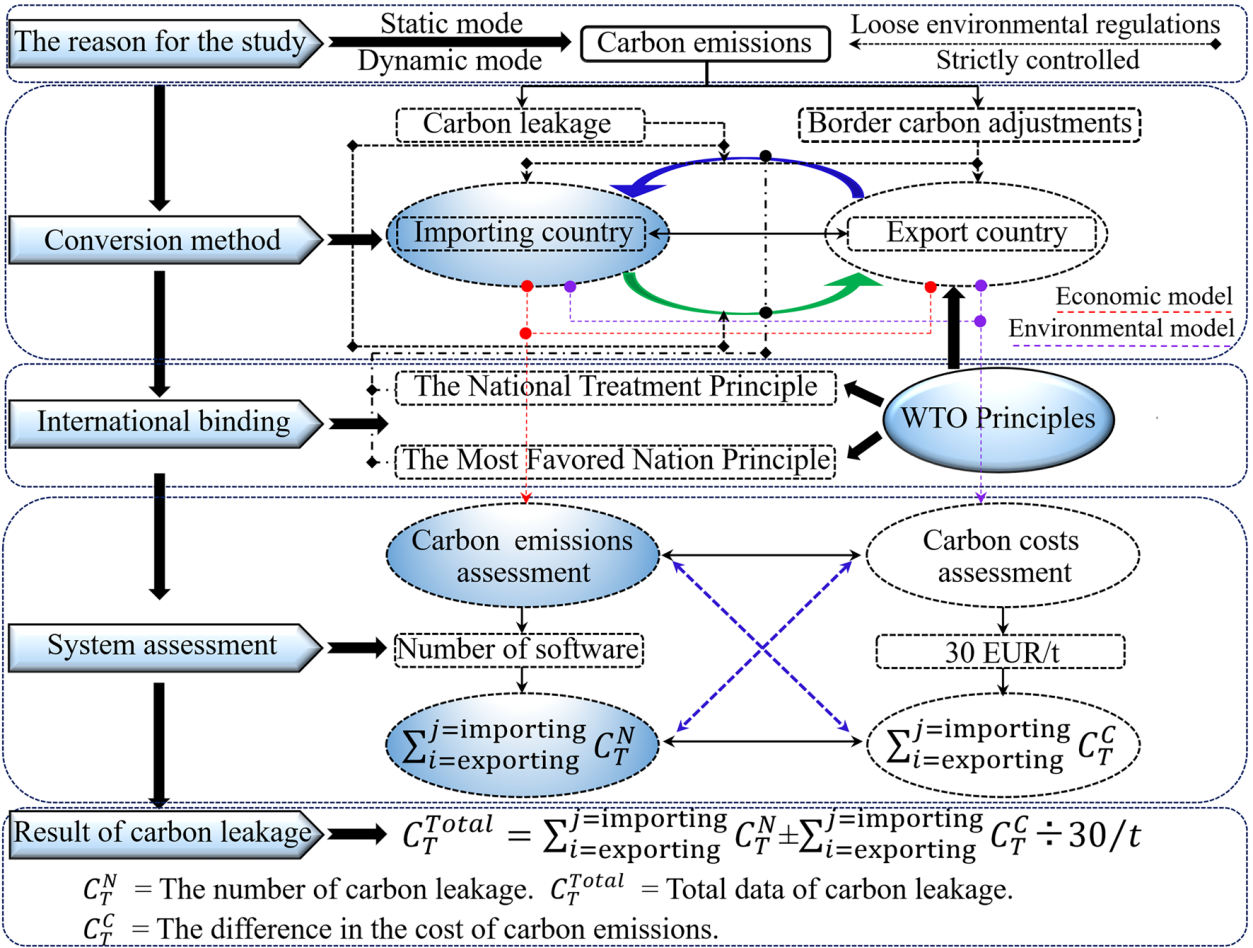
The matches model of carbon emissions.

Figure 3 shows the international investment project carbon trading system and carbon leakage model framework and analyzes the quota mode of carbon leakage in two ways: the first is that the carbon leakage amount of material products, personnel, and machinery is measured according to their carbon emissions (from essential material products → intermediate products → final products)^35,36^. The second is all costs and expenses incurred in the circulation and trading chain of goods after the finished products. They are exported to the international market through transportation channels and finally applied to the project construction (the carbon pricing is subject to the provisions of the EU Emission Trading System of EUR 30/t)^37^.

### Analysis of environmental input–output

Formula (1) (For (1)) model has been applied in the field of energy trade emissions for many years, mainly used to assess the environmental impact of national trade. The basic principle is to multiply the economic value of imported products by the environmental impact factor coefficient of the same products manufactured in the exporting country^38^.

1 $${P}_{m}= F\times {\left(I-A\right)}^{-1}\times T$$

${P}_{m}$ is $m$×$n$-order matrix, indicating that the environmental impact of Class $i$ is equivalent to the commodity export of $j$; $F$ is $m$×$n$-order matrix; $I$ is identity matrix; $A$ is the exporting country $n$×$n$ matrix; $T$ is exporting $n$×$n$ diagonal matrix according to commodity sorting.

### Industrial fuel-related CO_2_ emissions

Calculation formula of industrial fuel-related CO_2_ emissions:

2 $${C}_{i}= \sum \limits_{k=1}^{n}{P}_{ik}{\times V}_{k}{\times \upeta}_{k}, {T}_{i}=\frac{{C}_{i}}{{X}_{i}}$$

${T}_{i}$ is the CO_2_ emission intensity of industry $i$ (t/USD ~ United states dollar); ${X}_{i}$ is the total output value of sector $i$ (USD); ${C}_{i}$ is the CO_2_ emission directly generated by sector $i$ (t); ${\eta }_{k}$ is the CO_2_ emission factor of $K$ kinds of energy (t/T); ${P}_{ik}$ is the amount of $K$-type energy consumed by the region (t); ${V}_{k}$ is the conversion factor of $K$-type energy (T/t); $n$ is the number of energy types.

### Model of carton leakage

Based on the clarification of the concept of carbon leakage, Michalek and Schwarze discussed the calculation methods under different interpretations and policies^36^, which considered the inducement factors of strong and weak carbon emissions and environmental policy interference.

3 $${C}_{e}=\frac{{\Delta }_{c}\times {P}^{\alpha }}{{-\Delta }_{c}\times {P}^{\beta }}\times 100\mathrm{\%}$$

${C}_{e}$ is the carbon leakage amount (%); ${\Delta }_{c}$ is the carbon emission change data (kg);$p$ is the environmental constraint policy condition of carbon emissions; $\alpha$ is the countries, regions, and governments that implement carbon constraint policies; $\beta$ is the countries, regions and governments that have not implemented carbon constraint policies and whose carbon constraint is in a state of anarchy.

In Bangzhu Zhu's^36^ research, multiple formulas of implementation driving force and relative changes were added to the structural composition analysis, and formula (3) was transformed:

4 $${\text{ln}}{C}_{e}\left({t}_{1},{t}_{2}\right)=\sum_{i}{\text{ln}}{\left(\frac{{\overline{{\Delta }_{c}\times {P}^{\alpha }}}^{{t}_{2}}}{{\stackrel{-}{{-\Delta }_{c}\times {P}^{\beta }}}^{{t}_{1}}}\right)}^{{\overline{w} }_{i}}$$

The periods in different intervals of ${t}_{1}$ and ${t}_{2}$ in the formula; $i$ is the attribute parameter variable; ${\overline{w} }_{i}$ is the weighted share obtained in the attribute analysis in different time intervals.

#### Description of model

In Fig. 2, the eight carbon emission stages of the international investment project are analyzed. Among them, the first three are completed in the exporting countries; the two complete the fourth stage; the last four are completed in the importing countries. The carbon emissions at each stage are supervised according to the actual regional control policies. The regulatory constraints on production emissions and the application of production technologies are based on the national and regional framework. The current research aims to analyze the differences in carbon emission data in the process of carbon leakage and the establishment and optimization of the optimal carbon leakage model scheme and assessment system under the international investment project management background. In carbon trade, only the emission price of importing and exporting countries (the emission price is based on the regulatory process or the dynamic trade of the emission system market) is considered, and comprehensive analysis is carried out to improve the accuracy and effectiveness of the assessment. Zhou et al.^39^ studied the sustainable development of bridges in different regions of China. Their research process and theoretical model framework are similar to those used in international investment projects.

Calculation model of carbon emission at design stage:

5 $${C}_{{\text{contract}}}=\left\{\begin{array}{l}{C}_{{\text{design}}}={C}_{{t}_{1}}^{{\text{entry}}}+{C}_{{t}_{2}}^{{\text{exit}}}+{C}_{{t}_{3}}^{{\text{clearance}}}\\ {C}_{{t}_{1}}^{{\text{entry}}},{C}_{{t}_{2}}^{{\text{exit}}}={C}_{i} (\mathrm{entry \; or \; exit \; country})\\ {C}_{{t}_{3}}^{{\text{clearance}}}={P}_{m} (\mathrm{through \; the \; entry \; and \; exit \;country})\end{array}\right.$$

${C}_{{\text{design}}}$ is the carbon emission at the design stage (kg); ${C}_{{t}_{1}}^{{\text{entry}}}$ is the carbon emission of the exporting country (kg); ${C}_{{t}_{2}}^{{\text{exit}}}$ is the carbon emission of the importing country (kg); ${C}_{{t}_{3}}^{{\text{clearance}}}$ is the carbon emission during customs clearance (kg).

Calculation model of carbon emission at prepare stage:

The material cost accounts for about 60% of the total construction cost. Trade costs and carbon emissions increase due to the long destination of international investment projects, high transport costs, and customs clearance policy constraints of importing and exporting countries. Because of this, establishing a flexible material purchase and transportation network platform will improve supply efficiency and reduce economic costs, meet the material supply, and demand requirements and ensure the use efficiency.

6 $${C}_{{\text{prepare}}}\approx {C}_{i}$$

**Figure 4:**
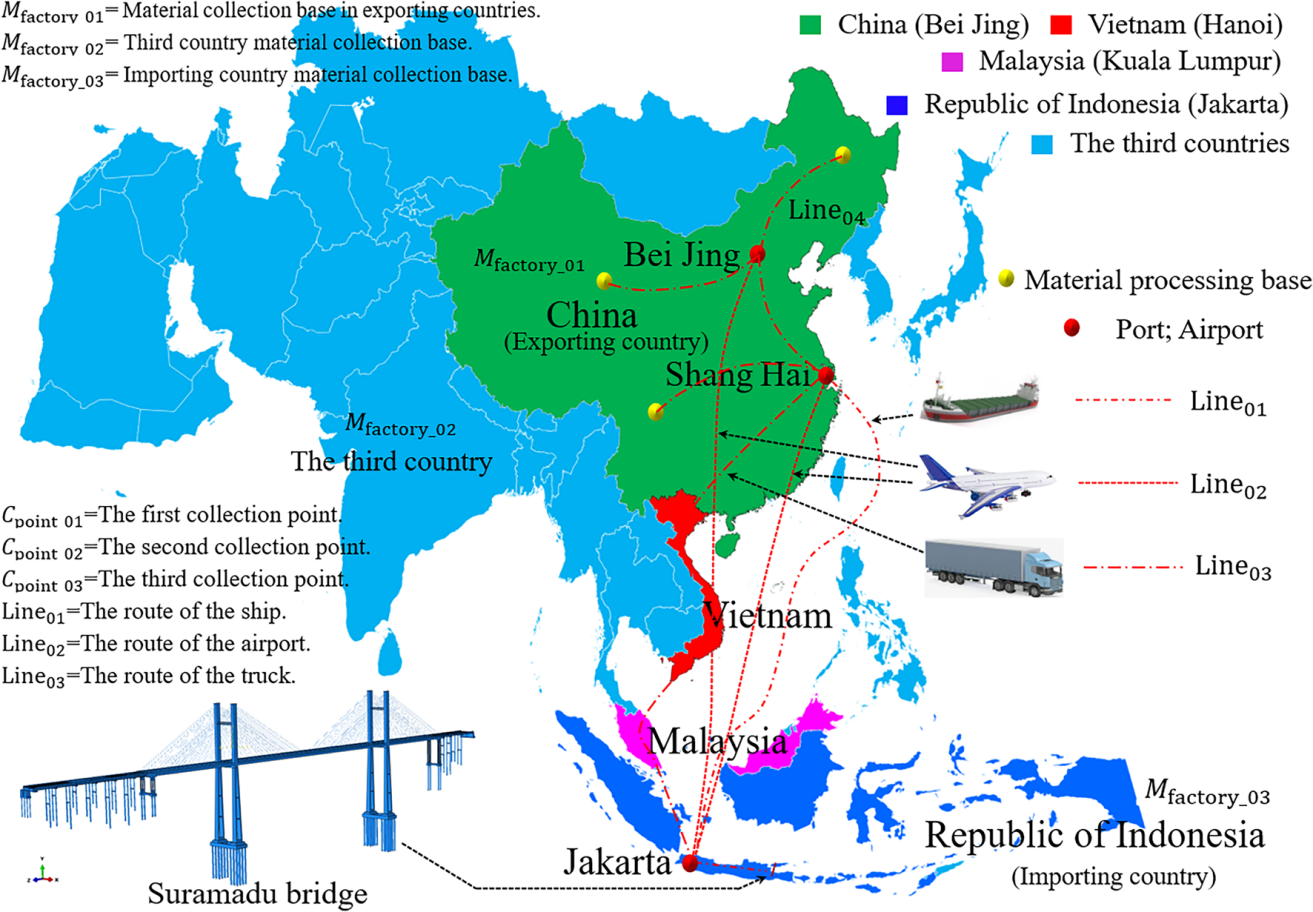
Models linked to material supply and transportation network platforms (Using Software Abaqus CAE).

The calculation model of carbon emission at the transport stage considers the following issues for the transport mode:

1. Measurement principles of project material management: ensure project cycle objectives, minimum cost, and best quality objectives. A multi-objective optimization algorithm model is established for material supply through coordination and compromise; the cooperation relationship between project material objectives is strengthened; the assimilation effect is produced; the optimal supply plan is obtained quickly^40^.
2. Establishment of a material supply and transportation network platform. There are three models for material procurement (For 7). The resource collection is set in three (> 3) ${M}_{{\text{factory}}\_0n}$ countries according to the geographical distribution (the first-level collection point), and the collected resources (the first-level collection point) are transported to the second-level collection point (set at the storage port for export) in batch. The third-level collection point is set in the importing countries (Fig. 4).
3. The carbon emission is calculated according to For (2).
  7 $$\left\{\begin{array}{ll}{M}_{{\text{factory}}\_01}\to {T}_{truck}^{line\_04}\to {C}_{{\text{point}}\_01}\to \left\{\begin{array}{l}{T}_{ship}^{line\_01}\\ {T}_{airplane}^{line\_02}\\ {T}_{vehicle}^{line\_03}\end{array}\right.\to {C}_{{\text{point}}\_03}\to {E}_{c}& \mathrm{The \; first \; model}\\ {M}_{{\text{factory}}\_02}\to {T}_{truck}^{line\_04}\to {C}_{{\text{point}}\_02}\to {C}_{{\text{point}}\_03}\to {E}_{c}& \mathrm{The \; second \; model}\\ {M}_{{\text{factory}}\_03}\to {T}_{truck}^{line\_04}\to {E}_{c}& \mathrm{The \; third \; model}\end{array}\right.$$

Calculation model of carbon emission at transport stage (Fig. 4):

8 $${C}_{{\text{transport}}}^{{\text{total}}}=\underset{{t}_{{\text{start}}}={{\text{Line}}}_{01}}{\sum^{{t}_{{\text{over}}}={{\text{Line}}}_{04}}}\left\{\left[{p}_{0}^{{{\text{Line}}}_{04}}\times {C}_{{p}_{4}}\times \left(1\pm {\lambda }_{{o}_{4}}\right)\right]+\left[{p}_{0}^{{{\text{Line}}}_{01}}\times {C}_{1}\times \left(1\pm {\lambda }_{{o}_{1}}\right)\bigcup {p}_{0}^{{{\text{Line}}}_{02}}\times {C}_{{p}_{2}}\times \left(1\pm {\lambda }_{{o}_{2}}\right)\bigcup {p}_{0}^{{{\text{Line}}}_{03}}\times {C}_{{p}_{3}}\times \left(1\pm {\lambda }_{{o}_{3}}\right)\right]\right\}$$

${p}_{0}^{{{\text{Line}}}_{01}},{p}_{0}^{{{\text{Line}}}_{02}}{,p}_{0}^{{{\text{Line}}}_{03}}{,p}_{0}^{{{\text{Line}}}_{04}}$ is the fuel consumption during material transportation (kg); ${C}_{{p}_{1}}{,C}_{{p}_{2}},{C}_{{p}_{3},}{C}_{{p}_{4}}$ is the carbon emissions of different oil plants (kg/kg); ${\lambda }_{{o}_{1}},{\lambda }_{2}{,\lambda }_{{o}_{3},}{\lambda }_{{o}_{4}}$ is the oil consumption rate (%).

Calculation model of carbon emission at customs clearance stage (ccs):

9 $$\left\{\begin{array}{ll}{C}_{ccs}^{1}=\underset{i={\text{entry}}}{\sum^{j={\text{exit}}}}\left\{{\alpha }_{entry}\times \left[{m}_{k}^{n}\times \left(1\pm {\lambda }_{k}\right)\times {c}_{k}\times \left(1\pm {\beta }_{c}\right)\right]-{\alpha }_{exit}\times \left[{m}_{k}^{n}\times \left(1\pm {\lambda }_{k}\right)\times {c}_{k}\times \left(1\pm {\beta }_{d}\right)\right]\right\}& \frac{{C}_{r}}{{C}_{r}}\\ {C}_{ccs}^{2}=\underset{i={\text{entry}}}{\sum^{j={\text{exit}}}}\left\{{\alpha }_{entry}\times \left[{m}_{k}^{n}\times \left(1\pm {\lambda }_{k}\right)\times {c}_{k}\times \left(1\pm {\beta }_{c}\right)\right]-{\alpha }_{exit}\times \left[{m}_{k}^{n}\times \left(1\pm {\lambda }_{k}\right)\times {c}_{k}\right]\right\}& \frac{{C}_{r}}{{C}_{f}}\\ {C}_{ccs}^{3}=\underset{i={\text{entry}}}{\sum^{j={\text{exit}}}}\left\{{\alpha }_{entry}\times \left[{m}_{k}^{n}\times \left(1\pm {\lambda }_{k}\right)\times {c}_{k}\right]-{\alpha }_{exit}\times \left[{m}_{k}^{n}\times \left(1\pm {\lambda }_{k}\right)\times {c}_{k}\right]\right\}& \frac{{C}_{f}}{{C}_{f}}\end{array}\right.$$

${C}_{ccs, }^{1}{C}_{ccs,}^{2}{ C}_{ccs}^{3}$ is to the carbon leakage amount (USD) after the adoption of carbon emission policy, which is bilateral or unilateral; ${C}_{r}$ is the unilateral carbon emission restricted areas; ${C}_{f}$ is the areas of carbon-free policy; ${m}_{k}^{n}$ is the material consumed in entering and exiting customs (kg); ${\lambda }_{k}$ is the loss rate of $k$ kinds of materials (%); ${c}_{k}$ is the carbon emission of $k$ kinds of materials (kg/kg); ${\alpha }_{entry}$ and ${\alpha }_{exit}$ are the carbon emission prices of the exporting and importing countries (USD/kg); ${\beta }_{c}$ and ${\beta }_{d}$ are the carbon quota for consumption materials provided by the exporting and importing countries (kg).

Calculation model of carbon emission at construction stage:

In the optimization framework of international investment project management, Zhou et al.^34^ analyzed the main factors affecting the sustainable development at construction stage: personnel, machinery, and materials. Under the constraints of different market environments, contractors can reduce project costs and improve project performance by adopting localized management model based on local human resources and cultural background characteristics. The common organizational structure of personnel in international investment projects is as follows: (1) Management personnel of the contracting country + skilled workers of the exporting country; (2) Management personnel of the contracting country + skilled workers of the exporting country + training workers of the importing country; (3) Management personnel of the contracting country + management personnel of the importing country + skilled workers of the exporting country + training workers of the importing country; (4) Management personnel of the contracting country + skilled workers of the exporting country + training workers of the third country; (5) Management personnel of the contracting country + management personnel of the importing country + skilled workers of the exporting country + training workers of the third country + training workers of the importing country (in general, management personnel of the contracting country: management personnel of the importing country = 1.53:1.00; skilled workers of the exporting country: training workers of the importing country = 0.66:1.00)^41^.

10 $${C}_{{\text{construction}}}=\underset{{t}_{{\text{prepare}}}={\text{start}}}{\sum^{{t}_{{\text{completed}}}={\text{end}}}}\left\{{P}_{{\text{number}}}\times {C}_{P}\times {T}_{{\text{duration}}}\times \left[\left(1\pm {\lambda }_{{\text{contracting}}}^{{\text{property}}}\right)\bigcup \left(1\pm {\lambda }_{{\text{importing}}}^{{\text{property}}}\right)\bigcup \left(1\pm {\lambda }_{\mathrm{third country}}^{{\text{property}}}\right)\right]\right\}+{C}_{{\text{transport}}}^{{\text{total}}}+{C}_{i}$$

${P}_{{\text{number}}}$ is the average number of people during the construction (people); ${C}_{P}$ is the carbon emission per capita (kg/day/people); ${T}_{{\text{duration}}}$ is the effective working duration (day); ${\lambda }_{{\text{contracting}}}^{{\text{property}}}, {\lambda }_{{\text{importing}}}^{{\text{property}}}{, \lambda }_{\mathrm{third country}}^{{\text{property}}}$ is the carbon emission loss rate under the three models (%).

Calculation model of carbon emission at trial operation stage:

At this stage, relevant units and service operators generally test and assess the multimodal transport system of the completed project to determine whether the project quality and various design index data are qualified^42^.

11 $${C}_{\mathrm{trial \;operation}}\approx {C}_{{\text{construction}}}$$

Calculation model of carbon emission at maintenance stage: Maintenance and overhaul service is one of the key strategies to extend the equipment life cycle. In addition to the material life cycle specified in the specifications, the system complexity degradation shall also be checked and monitored. Three key factors need to be verified first during maintenance: redundancy, repeatability, and control^43^.

12 $${C}_{\mathrm{maintenance }}={C}_{{\text{construction}}}\times \frac{{T}_{{\text{design}}}^{{\text{usage}}}}{{T}_{{\text{regulation}}}^{\mathrm{replacement\; time}}}\times \left(1\pm {\lambda }_{r}\right)\times \left(1\pm {\zeta }_{m}\right)$$

${\lambda }_{r}$ is the change rate of carbon emissions increase and decrease caused by road closure, diversion and temporary measures taken during maintenance (%); ${\zeta }_{m}$ is the compensation and correction coefficient obtained after analyzing the project monitoring and assessment data (0.00–1.00).

Calculation model of carbon emission at handover finish stage:

13 $${C}_{{\text{handover}}}\approx {C}_{{\text{construction}}}$$

≈ indicates that the analysis model at this stage is similar to the construction stage.

#### Analysis of model

According to the model frameworks in Figs. 2 and 3, the international investment project carbon emission assessment model is established in Section Model of carton leakage, and the theoretical model for assessment in this paper is obtained after integrating eight stages:

14 $${C}_{Total}=\underset{T={\text{start}}}{\sum^{T={\text{end}}}}\left(\begin{array}{l}{C}_{{\text{contract}}}+{C}_{{\text{prepare}}}+{C}_{{\text{transport}}}^{{\text{total}}}+{C}_{ccs}^{n}+{C}_{{\text{construction}}}+{C}_{\mathrm{trial \; operation}}+\\ {C}_{\mathrm{maintenance }}+{C}_{{\text{handover}}}\end{array}\right)$$

Both ${C}_{{\text{contract}}}$ and ${C}_{ccs}^{n}$ have carbon leakage.

## Results

**Figure 5:**
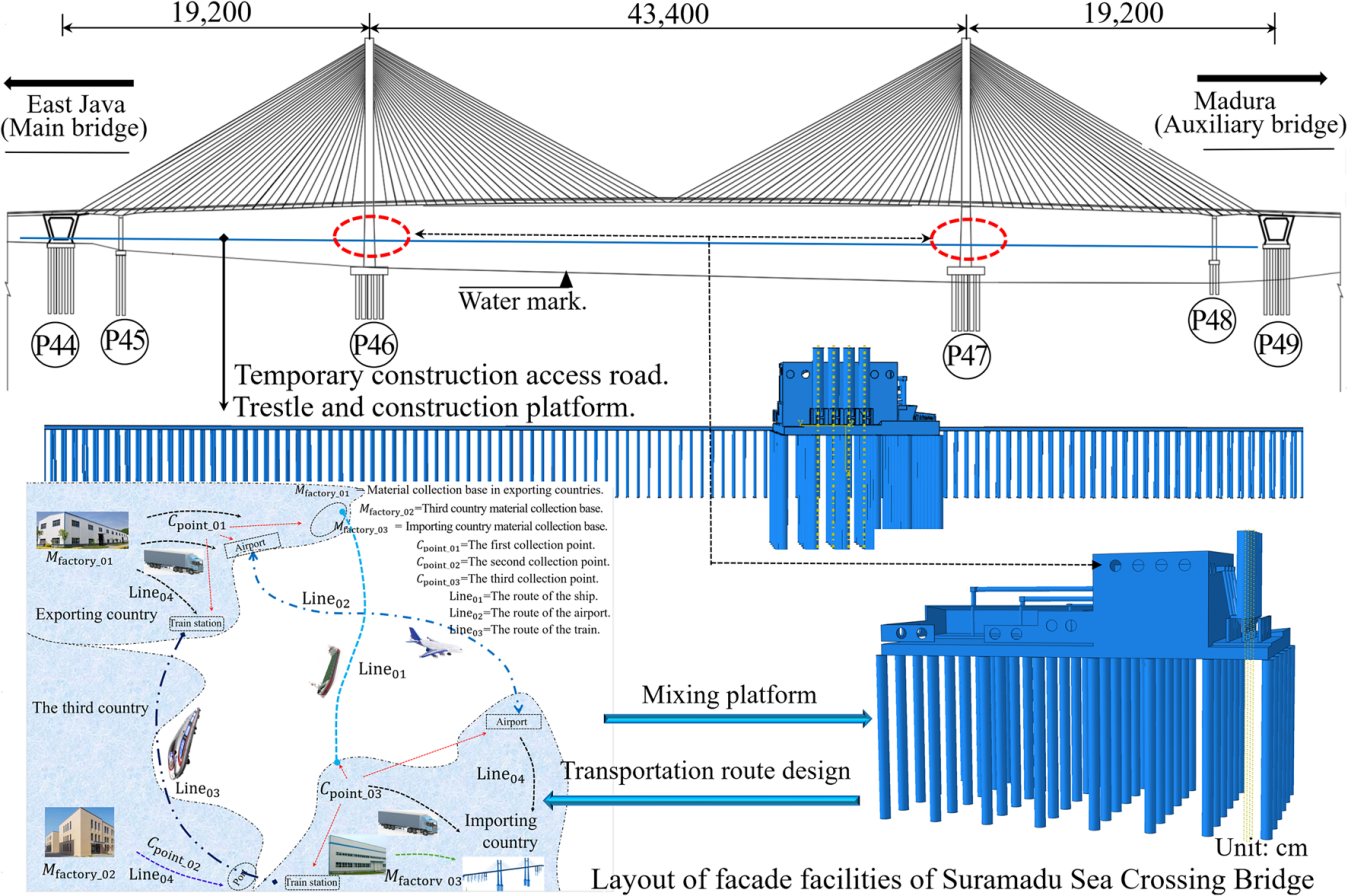
The façade and auxiliary facilities layout of the SSCB (Using Software Abaqus CAE and AutoCAD2022).

Suramadu Sea-Crossing Bridge (SSCB) is located in Jawa Timur, Indonesia, and crosses the Madura Strait, an international cooperation project invested in, designed, and constructed by the Chinese government under the EPC model (Figs. 4, 5). The bridge’s total length is 5.4km, and the main bridge is an 818m (192 + 434 + 192 m) cable-stayed bridge with twin pylons and twin cable planes. The auxiliary bridge is a 2 × 672 m (40 + 7 × 80 + 40 + 32 m) continuous box girder bridge.

The main bridge adopts a two-way four-lane design, with motor vehicle loading of the road—Level I; design speed of 80 km/h; design basic wind speed of 27.0 m/s; seismic horizontal acceleration peak of 0.15–0.24 g; temperature action of 20–45 ℃; navigation clearance of 400 m × 35 m. The main beam box girder is assembled by welded box-type steel box girder, divided into 73 beam sections (standard beam section is 12 m). The box girder is transported to the site for lifting and installation after processing at the factory (Fig. 5). The cable tower is H-shaped, and the main tower is 141.33 m high. The QPM-50 hydraulic climbing formwork is used for 33 times of sectional pouring, and the bridge foundation is constructed by bored piles with R of 1.8–2.4 m and pile length of 70–104 m. The bearing platform of the main bridge is 57.2 m × 34 m in size, with a thickness of 6 m^44^.

### Analysis of carbon emission

According to Figs. 2 and 3, the model framework is established, and the design documents, construction data and published research documents are used to analyze the carbon emissions and leakage at each stage.

### Design and prepare stage

SSCB is designed by the turnkey company by the *Highway Bridge Design Code* (JTG D60-2004), with a design reference period of 100 years. According to the organization mode of international investment project survey and design, the project is divided into the investigation stage (including the organization of early stage and logistics personnel, preliminary site investigation, operation condition survey, and communication with an investor) → planning stage (including the establishment of project organization structure, preparation of project plan and exit preparation and handling) → implementation stage (including project organization management and logistics support) → closeout stage (including the compilation of original data and professional data and handover, equipment and personnel evacuation)^45^.

The work during the design mainly includes geological survey and exploration, which is planned to complete in 3.6 months. The operation methods include drilling exploration of the sample, geophysical prospecting of wave velocity, and field tests. The carbon emissions at the design stage are concentrated on transportation (and), mechanical oil consumption, power consumption of surveying and mapping equipment, and energy consumption of personnel during interior and fieldwork^39^. ${Line}_{02} \;\; \mathrm{ and} \;\; {Line}_{03}$ the fieldwork is completed in the engineering area of Surabaya, and the collected data are sent back to the design company through the transmission equipment. The design personnel complete all design work according to the geological survey and image data. The actual productivity in different regions and dimensions is determined by the changes in the working environment and the organizational performance at the design stage^46^. As the environmental impact of the survey work is far more significant than that of the indoor design, the carbon emission analysis data are included in the carbon emission range of the importing country.

**Figure 6:**
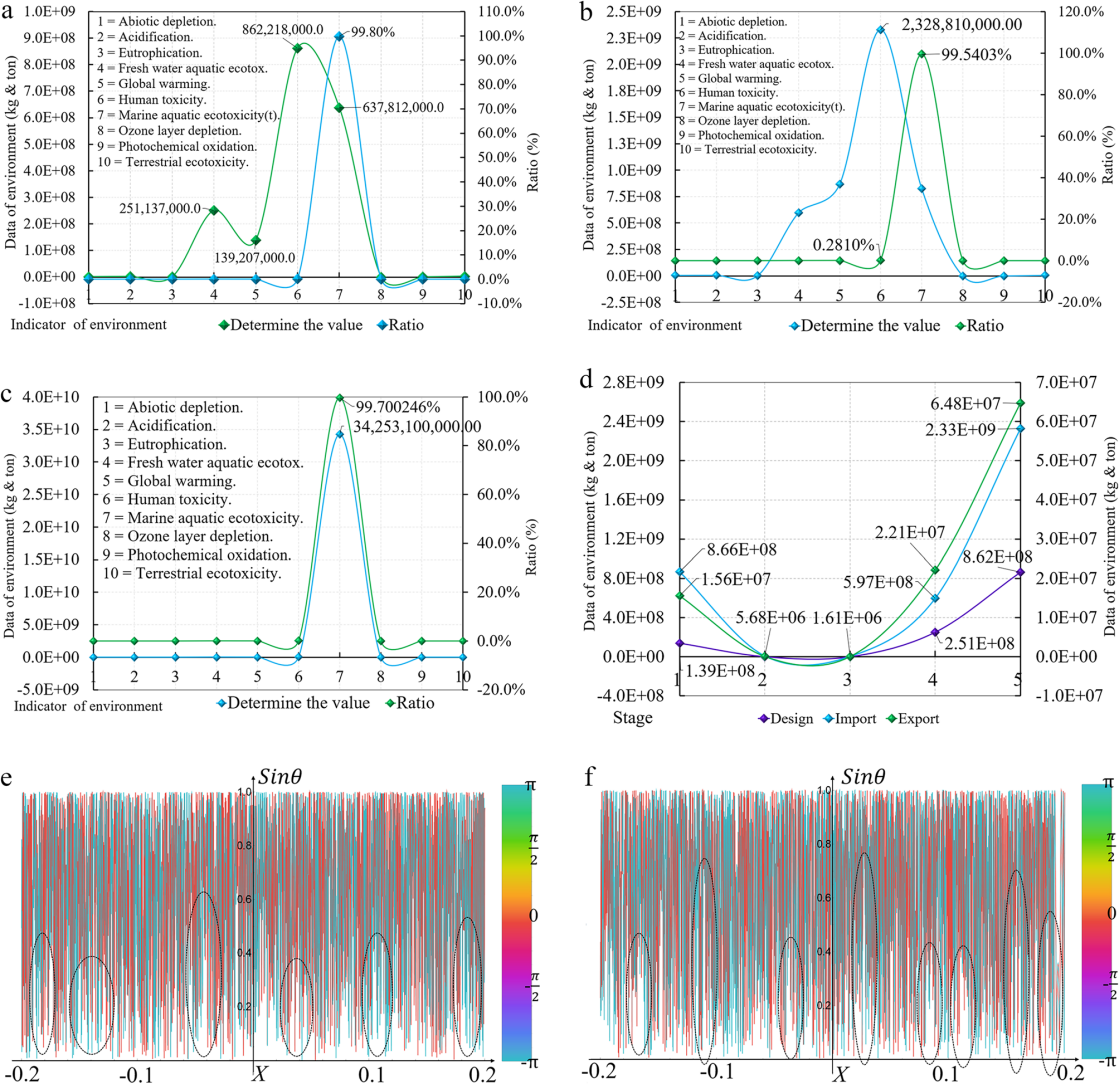
Impact data of SSCB. (**a**) Environmental impact data of design stage. (**b**) Environmental impact data of import stage. (**c**) Environmental impact data of export stage, (**d**) Comparison of five groups of environmental impact data. (**e**) Global warming impact data of math model. (**f**) Human toxicity impact data of math model.

During the data analysis, the modeling software openLCA1.10 is used for the calculation, showing that the total emission at the design stage is 639.07 million tonnes (t), of which marine aquatic ecotoxicity accounts for 99.80% of the total, and the global warming (GWP100) emission is 139.21 thousand t, accounting for 0.022% of the total (Fig. 6a).

The model in Fig. 2 shows the work content at the preparation stage, and the material consumption, transportation, and equipment energy consumption of SSCB. According to the assumptions in Section Methodology, concrete, bellows, and auxiliary steel are all purchased in the importing country. The bridge is located in the equatorial oceans, with high sunshine temperatures, significant evaporation of seawater, and severe damage and corrosion to the structure caused by air humidity, so structural durability is the core index of material selection^47^. However, some materials in the importing country need to meet the specification requirements, so the steel strand stays in the exporting country and provides cable, anchorage, 9and reinforcement. As shown in Fig. 6b, the emissions of the importing countries are 82.87 million t, and the global warming emission is 866.09 thousand t, accounting for 0.105% of the total. As shown in Fig. 6c, the emissions of the exporting countries are 34.35 million t in total, and the global warming emission is 15.56 thousand t. As shown in Fig. 6d, the data curve of the five largest groups of influencing factors at three different stages changes in a quadratic parabola. Through the analysis of the mathematical model established by Wolfram Mathematica, there is an uninterrupted asymmetric cycle curve in the interval (− 0.2–0.2), which proves that the carbon leakage in the importing and exporting countries and at the design stage shows a discontinuous curve, and the peak and valley values occur continuously. $\mathit{sin}\theta$ the carbon leakage in the importing countries is higher than in the exporting countries (Fig. 6e,f).

### Transport and customs clearance stage

**Figure 7:**
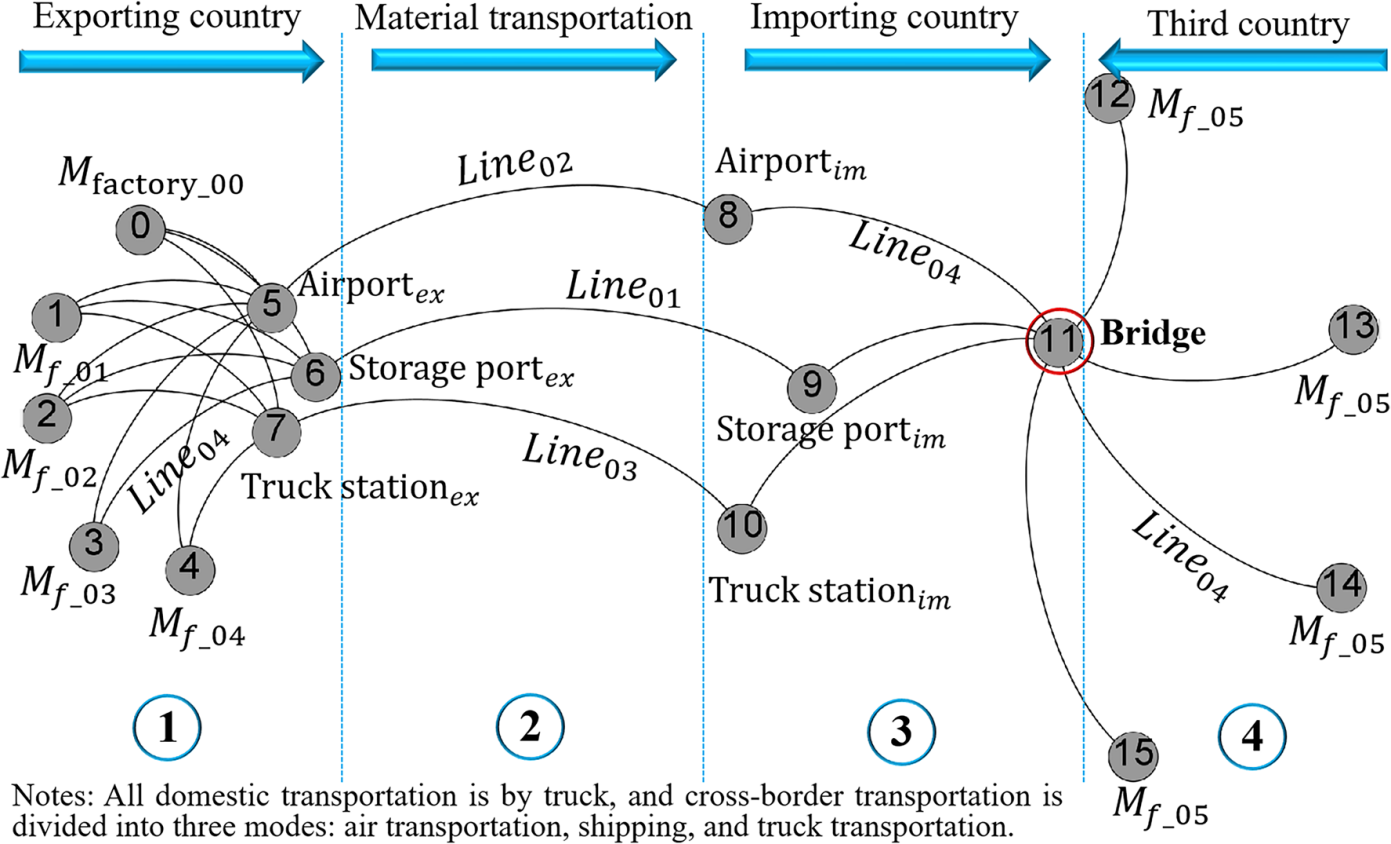
Supply chain logistics transportation network model of SSCB.

In Figs. 2, 3, and 4, the mathematical model and emission path of carbon leakage at the transport stage are analyzed. The transport mode framework is divided into three parts: (1) Storage of exporting countries' materials at the port or airport freight yard respectively for transportation (${Line}_{04}$); (2) Supply chain business logistics transportation from the exporting countries to the importing countries (${Line}_{01,}{Line}_{02,}{Line}_{03}$); (3) Transportation from the port or airport of the importing countries to the project site of Surabaya (${Line}_{04}$);transportation from the third country to the project site (${Line}_{04}$). In Figs. 4 and 7, three logistics chain transportation models (optimization criteria: minimizing cost and environmental impact) are designed for the transportation mode. The shipping frequency and vehicle type are selected according to the dynamic model of multimodal transport^48^.

Through the analysis in 4.1.1, five groups of impact categories are selected as the core environmental impact factors of fossil materials to improve the clarity of research ideas and data robustness: global warming potential (GWP), acidification potential (AP), free-water eutrophication potential (FEP), particulate matter formation potential (PMFP), including fumes and dust, and waste potential (WP)^49^.

According to the data analysis, the environmental impact emission in the ① interval (Fig. 7) is 25.91 t, GWP = 25.776 t, accounting for 99.48% of the total. ② interval is divided into three transport modes, and ${Line}_{02}$ main mode is air transportation. The main influencing factors are monetary cost and service quality. The time cost includes control time such as cruise, loading and unloading, customs clearance time and flight delay, etc. The average handling control time of the international airport is five h. The aircraft type used is Boeing 747–400 with a total takeoff weight of 395 t (empty weight of 178.80 t; fuel consumption of cargo is 10 t/h), and the cruise time is 9.75 h (Shanghai Pudong International Airport to Sukarno International Airport)^49^. According to the research model, the materials purchased by the exporting country are stored in the Port of Shanghai, and all transnational transport materials are delivered here.

${Line}_{01}$ For international cargo shipping, the port of departure is the Port of Shanghai, and the port of discharge is the Port of Surabaya, Indonesia. The journey is 2267 km, and the regular shipping time is 12 d. green container shipping is adopted (international standard: 45-foot-high container; 25 t; speed: 22.3 knots; 0.0512 t per twenty-foot equivalent unit per day)^50^.

${Line}_{03}$ truck transportation starts from the distribution center of Port of Shanghai, and the route is China → Laos → Thailand → Malaysia → Singapore → Indonesia (Jakarta). The journey is 7567 km, and the driving time is 135 h. The truck is 17.5 m, and the load is 30 t^51^.

According to the design model in Fig. 2, asphalt, concrete, bellows, and angle steel are all purchased from importing countries and third countries. The concrete of SSCB is new high-strength concrete, which the General Contractor produces in the mixing plant built at the project site, and all raw materials are purchased from the importing countries^52^.

**Figure 8:**
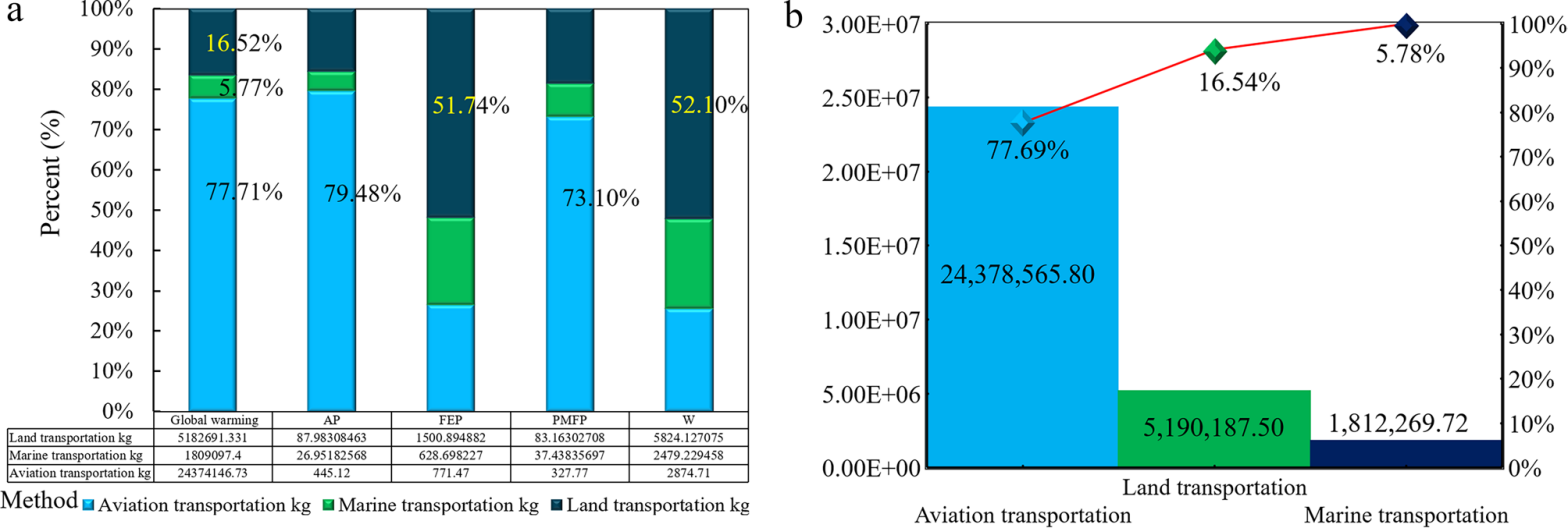
Supply chain logistics transportation network environmental impact assessment of SSCB. (**a**) Environmental impact data under three transportation modes. (**b**) Comparison of total environmental impact data under three transportation modes.

The total emissions at the transport and customs clearance stage shown in Table 1 and Fig. 8 under three different modes are 24,378,565.80 t, 1,812,269.72 t, and 5,190,187.50 t, respectively. According to the data analysis, the environmental impact emission under the shipping mode is the lowest, accounting for 7.43% of the total, while truck transportation accounts for 34.92%.

**Table 1:** SSCB's supply chain logistics and transportation environmental impact data.

Region	Lines		Unit	Global warming	AP	FEP	PMFP	W
Exporting	1	Line04	kg	25,775.96	0.12	26.56	1.10	105.97
Transportation + third	2 + 3	Line02	kg	23,863,300.99	442.27	188.22	306.90	560.85
	2 + 3	Line01	kg	1,298,251.66	24.10	45.45	16.57	165.36
	2 + 3	Line03	kg	4,671,845.59	85.13	917.65	62.30	3,510.26
Importing + third	4	Line04	kg	485,069.78	2.74	556.68	19.77	2,207.89

### Construction stage

The carbon emissions of SSCB design materials are analyzed in Results, and the environmental impact emission generated during the construction is mainly studied. According to the construction organization design and published literature analysis, the construction plan is adopted for essential structural parts. (1) The φ80 cm steel pipe pile foundation is adopted for the erection of the offshore construction platform, and the top plate is connected by the Bailey truss and steel plate (Fig. 5). (2) Two HZS90 offshore concrete centralized mixing stations will be built. Equipped with transport ships and lifting ships as raw material transportation and lifting machinery (Fig. 5). (3) The main beam is assembled with a steel box girder, and the hoisting system is used to complete the offshore assembly. The 0 # and one # main beams are constructed with the scaffolding. (4) The main tower is constructed with sliding formwork, and the 44 # and 49 # V-shaped piers are constructed with hydraulic climbing formwork. (5) All pile foundations are constructed with a fixed platform, and steel casing is embedded in advance to complete the grouting work with ZSD250/150 drilling rig^53^. The above equipment and materials are all auxiliary facilities purchased in the importing countries and third countries, considering the principle of economical transportation.

As shown in Table 2, the emission at this stage is 12,691,275.98 t, of which global warming accounts for 88.46%; W accounts for 6.60%; PMFP accounts for 3.08%.

**Table 2:** SSCB's construction stage environmental impact data.

Name (kg)	Global warming	AP	FEP	PMFP	W
HZS91 mixing station	607,155,445.45	7,862,401.29	4,881,237.67	21,090,164.11	45,237,374.16
V-shaped pier column	270,225,878.39	3,489,408.72	2,172,352.28	9,360,060.59	20,100,069.18
Trestle platform	9,973,859,519.73	129,442,760.35	80,284,532.34	347,217,700.35	744,464,753.97
Main tower slip form system	375,584,260.25	4,857,778.03	3,014,652.32	13,030,516.97	27,945,111.86

### Trial operation; maintenance and handover stage

SSCB was officially completed and opened to traffic in June 2009. The main beam of the cable-stayed bridge is anchored and connected by a steel structure. Moreover, the internal and external surfaces of the steel beam are coated with the anti-corrosion coating (using the arc spraying zinc + aluminum composite coating), with a design life of more than 20 years^54^. By 2022, the bridge will have been in operation for 13.5 years, and the main structural elements do not need to be replaced. According to JTGB01-2014, the regular service life of asphalt pavement for the expressway is 15 years^55^, and no replacement is required during the maintenance. Therefore, the main environmental impact of the two stages is the carbon emissions of maintenance personnel and vehicle equipment.

The handover stage is the critical transition period between construction and regular operation. After completion, the project and the whole construction process data shall be submitted according to the contract. According to the contract’s one-year defect warranty period, the contractor will retain some management personnel and technical workers for maintenance. Therefore, there will still be a small part of material and energy consumption, and the principle of nearby procurement is still adopted to reduce costs^56^. The damaged particular parts are purchased from the country of origin (exporting country), and the impact of carbon leakage will not be included due to the limited quantity.

As shown in Table 3, a total of 25,961.10 t was emitted in the two stages. Of which global warming accounted for 13.75%, 79.04%, and 5.35% of the total. The time interval includes two years of trial operation, 11.5 years of management, and one year of handover analysis.

**Table 3:** SSCB's trial operation and management stage environmental impact data.

Number (Ton)	Global warming	AP	FEP	PMFP	W
Trial operation	3568.73	0.00	14.04	0.17	54.64
Management	20,520.20	0.01	80.73	0.98	314.19
Handover stage	1,387,734.99	1.92	3994.30	65.36	15,604.82

## Discussion

The environmental impact data in Figs. 2, 3, and 4 were assessed in Section Conclusion. According to the research objective, the optimized economic indexes of carbon leakage and emissions of international investment projects should be determined finally, according to the principle that the transport cost has been included in the EPC contract in the project bidding. Moreover, it is considered according to the transaction cost model of the construction project^57^, and the ② interval (material translation) in Fig. 7 is included in the carbon emission data sequence of the importing country.

### Carbon emission analysis of stage

**Figure 9:**
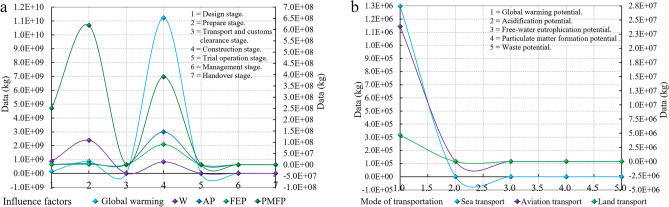
Data environmental impact assessment of SSCB. (**a**) Emissions in seven stages. (**b**) Environmental impact emissions under three different transport modes.

According to the model in Fig. 2, it is divided into eight stages. Transport and customs clearance are combined into data analysis stages because they are carried out simultaneously. According to the analysis data, the three types of transport emissions in the second zone in Figs. 7 and 9b are 23,864.80 t for air transportation; 1298.503 t for shipping; 4676.42 t for truck transportation, namely 18.38:1.00:5.10, so shipping is the most environmentally friendly transport mode. Figure 9a shows that the environmental impact data in the seven stages is Design stage = 1,255,330.28 t. Prepare stage = 3,901,530.27 t; Transport and customs clearance stage = 1812.27 t; Construction stage = 12,691,275.98 t; Trial operation stage = 3637.59 t; Management stage = 20,916.11 t; Handover stage = 1407.40 t.

The environmental impact data at the construction stage ranks first, accounting for 71.00% of the total. The reasons are (1) Complex construction environment conditions, particularly climate and hydrology. (2) Long construction period, namely, 1334 days from commencement to completion, consuming a lot of workforces and mechanical lifting equipment. (3) Impact of navigation, increasing the construction safety control risk and facility investment to ensure standard waterway navigation. (4) Additional ancillary works during offshore operations, consume a large amount of steel and concrete, and increase carbon emissions. (5) Requirements of different national boundaries for dispatched workers in international investment projects (the fifth organizational personnel model in For (10)). The local workers are unskilled technicians, and the working hour is implemented in strict accordance with 6 h, which has seriously affected the construction progress and flow operation.

### Carbon leakage and expense analysis of stage

**Figure 10:**
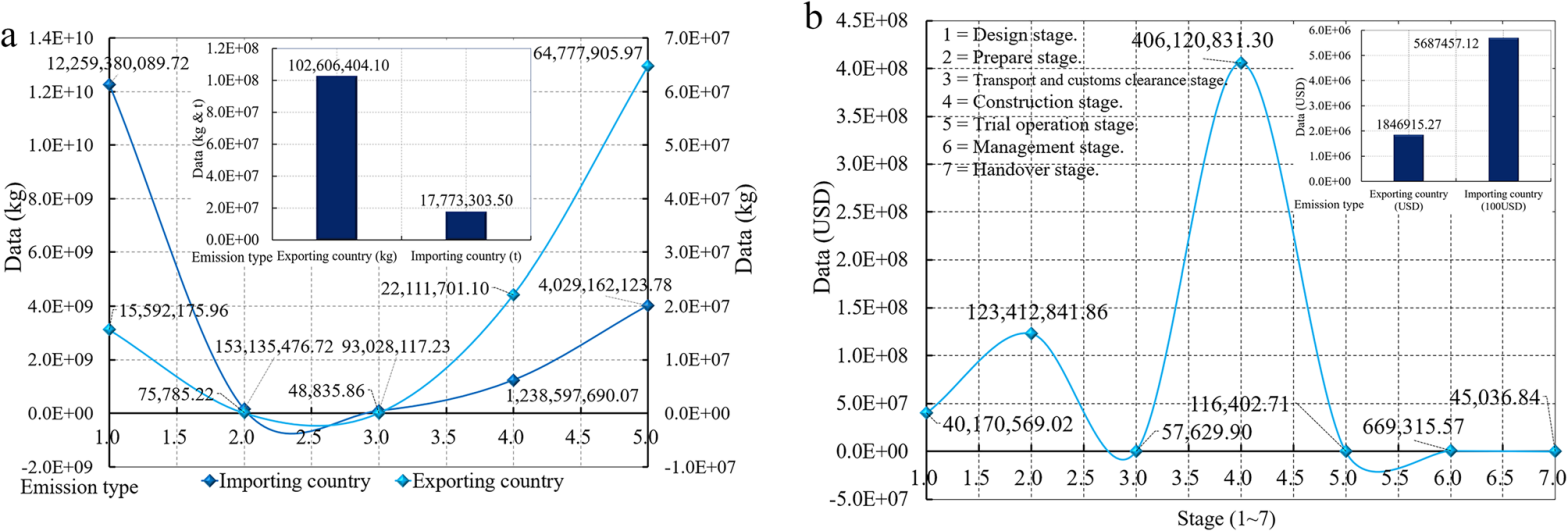
Data carbon emission of SSCB. (**a**) Emissions of type. (**b**) Transaction value of type.

As shown in Fig. 10a, the total emission of exporting countries and importing countries is 102,606.40 t and 17,773,303.50 t, with the ratio of 5.77:1000. The economic change of carbon trading is analyzed based on the value of 18USD/t according to the trade price of carbon emission in China (exporting country) of 100–120CNY/t (14–20USD)^58^. The economic change of carbon trading of the importing country is analysed according to the EU market price. The economic and trade quota generated by the total emissions of SSCB is 570,592,627.19USD, of which the trade quota of the exporting country is 1,846,915.27USD, and the trade quota of the importing country is 5,687,457.12USD, with the ratio of 3.25:10 (Fig. 10b).

## Conclusion

With the frequent exchanges of international trade, countries worldwide are controlling and reducing the impact of carbon leakage and carbon trade on their sustainable development through laws, regulations, and tariff policies. The literature survey found that accurate data analysis and coupling evaluation of research models need to be further improved and supplemented by researchers.

This paper analyses the mechanism and influencing factors of international projects' carbon emissions and carbon leakage through the theoretical model of the carbon leakage research framework. The research discussed optimizing and evaluating participating countries' carbon leakage data and carbon trade quota. The analysis data after the study shows that the ratio of carbon leakage and emissions in each stage of international engineering projects is: 1,255,330.28: 3,901,530.27: 1812.27: 12,691,275.98: 3637.59: 20,916.11: 1407.40 t. Carbon leakage is mainly concentrated in the three stages of design, preparation, and construction, and the ratio is 1.00:3.11:10.11. Therefore, environmental control during the construction stage is very critical. At the same time, the trade quota between the exporting and importing countries is 3.25:10.00, and the importing country has to pay more funds to deal with the trade balance.

This study's theoretical model and case analysis can clearly understand the process of carbon leakage and carbon trade generated in international engineering projects, providing a paradigm for research in this field. The theoretical model in this paper only applies to the carbon footprint data assessment of international engineering projects in the construction industry. Because of the uncertainty and dispersion of the influencing factors, it cannot represent all international trade industries.

The future research direction is how to evaluate the construction industry's carbon leakage and carbon trade data in the global international trade; this will be challenging and important work, especially for accurately assessing and dividing the global carbon leakage path.

## Data Availability

All data generated or analysed during this study are included in this published article.
